# Supplementary material for: Alpha-Tocopherol Metabolites (The Vitamin E Metabolome) and Their Interindividual Variability during Supplementation
Source: Antioxidants (Basel). 2021 Jan 25;10(2):173. doi: 10.3390/antiox10020173 (PMC7912187; doi:10.3390/antiox10020173)
Supplement: Supplementary file 1 [file antioxidants-10-00173-s001.pdf]

Suppl. Tables

**Supplementary Table S1. General characteristics of the healthy subjects investigated in this study.**		
	Male	Female
Number	7	10
Age	37.2±9	31.9±7
Weight (kg)	71.2±7	56.2±8
BMI	22.7±2	20.1±2
Waist circumference (cm)	83.5±7	69.5±7.8

Supplementary Table S2. Correlations between vitamin E metabolomics data and subjects age and anthropometric parameters.

Correlation **Age** vs	pre		post	
	R^2	p-value	R^2	p value
α-TOH	0.28	**0.02**	0.13	0.15
γ-TOH	0.00	0.82	0.00	0.94
α-TQ	0.00	0.99	0.04	0.37
α-13'COOH	0.08	0.25	0.03	0.49
M1	0.02	0.49	0.02	0.52
M2	0.19	0.07	0.01	0.66
α-13'OH	0.06	0.29	0.07	0.28
M3	0.04	0.41	0.07	0.26
α-CMBHC	0.05	0.33	0.00	0.84
α-CECH	0.00	0.74	0.10	0.22
γ-CECH	0.00	0.85	0.00	0.92

TableS3

Correlation **BMI** vs	pre		post	
	R^2	p-value	R^2	p value
α-TOH	0.05	0.36	0.02	0.53
γ-TOH	0.01	0.68	0.14	0.13
α-TQ	0.04	0.40	0.02	0.51
α-13'COOH	0.00	0.75	0.01	0.59
M1	0.17	0.09	0.14	0.12
M2	0.14	0.12	0.25	**0.03**
α-13'OH	0.05	0.38	0.03	0.48
M3	0.04	0.43	0.20	0.06
α-CMBHC	0.41	**0.005**	0.08	0.24
α-CECH	0.03	0.50	0.20	0.08
γ-CECH	0.01	0.68	0.14	0.13

Table s4

Correlation **WC** vs	pre		post	
	R^2	p-value	R^2	p value
α-TOH	0.17	0.09	-0.00	0.71
γ-TOH	0.06	0.33	0.03	0.49
α-TQ	0.04	0.40	0.00	0.92
α-13'COOH	0.01	0.67	0.04	0.39
M1	0.19	0.08	0.10	0.20
M2	0.15	0.12	0.26	**0.03**
α-13'OH	0.01	0.61	0.02	0.51
M3	0.06	0.30	0.29	**0.02**
α-CMBHC	0.58	**0.00**	0.04	0.40
α-CECH	0.04	0.43	0.04	0.45
γ-CECH	0.06	0.33	0.03	0.49

Table S5

Supplementary Table S3. Multiparameter regression analysis of the different metabolites and absolute and cholesterol-corrected leves of α-TOH levels determined before and after supplementation

Correlation	α-TOH pre		α-TOH post	
	R^2	p-value	R^2	p value
γ-TOH	0.01	0.69	0.06	0.32
α-TQ	0.00	0.86	0.25	**0.04**
α-13'COOH	0.03	0.45	0.07	0.28
M1	0.18	0.09	0.04	0.39
M2	0.02	0.54	0.00	0.79
α-13'OH	0.18	0.08	0.05	0.34
M3	0.00	0.95	0.16	0.10
α-CMBHC	0.08	0.25	0.00	0.81
α-CECH	0.01	0.61	0.01	0.63
γ-CECH	0.01	0.69	0.06	0.32

Correlation	α-TOH/Cholesterol			
	Pre		Post	
	R^2	p	R^2	p
γ-TOH	0.02	0.52	0.00	0.93
α-TQ	0.08	0.26	0.04	0.43
α-13'COOH	0.19	0.07	0.15	0.11
M1	0.48	**0.001**	0.01	0.63
M2	0.06	0.33	0.02	0.51
α-13'OH	0.09	0.21	0.05	0.34
M3	0.04	0.42	0.00	0.89
α-CMBHC	0.00	0.78	0.00	0.87
α-CECH	0.09	0.22	0.19	0.08
γ-CECH	0.07	0.29	0.11	0.18

Supplementary Table S4. Levels (nM) of plasma PUFA in healthy volunteers before (Pre) and after (Post) supplementation with α-TOH

	Pre		Post	
	Mean	SD	Mean	SD
20-COOH AA	14	4.6	21.8	8.9
EPA	365	382.9	395	346.9
AA	1242	518.8	2046	1177.6
DHA	1825	994.2	2634	1527.1
ALA+GLA	2156	1006.2	3537	1705.9

**Supplementary Table S5. Regression analysis coefficients (R^2) of absolute and cholesterol-corrected levels of α-TOH and PXR measured before (Pre) and at the end (Post) of the supplementation protocol.**			
		PXR	
		Pre	Post
α-TOH	Pre	**0.489**	**0.322**
	Post	0.287	0.066
α-TOH/Cho	Pre	**0.321**	**0.414**
	Post	0.179	0.091

**Supplementary Table S6. Regression analysis coefficients (R^2) of M1 metabolite and PXR measured before (Pre) and at the end (Post) of the supplementation protocol.**			
M1	Pre	0.105	0.183
	Post	0.117	**0.295**

Suppl. Figures

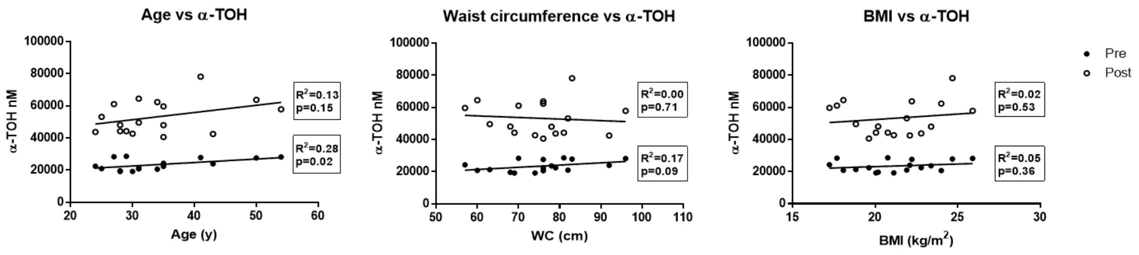

Supplementary Figure S1. Correlation between α-TOH and age (A), WC (B) and BMI (C) of healthy subjects studied before (pre) and after (post) supplementation with RRR-α-TOH. *$p < 0.05$.

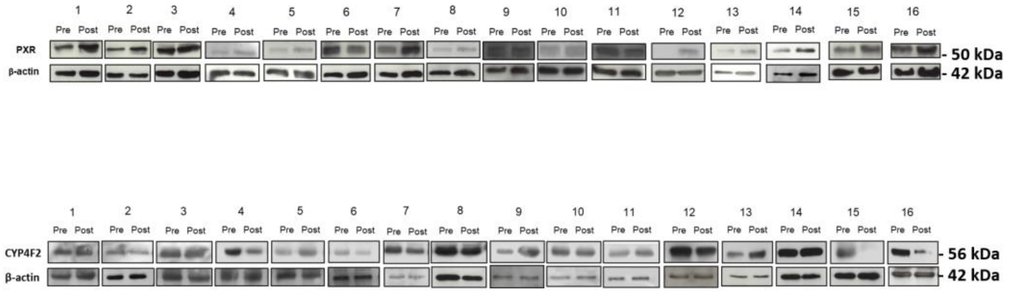

Supplementary Figure S2. PXR and CYP4F2 protein expression in PBMLs of healthy subjects measured by immunoblot before (pre) and after (post) α-TOH supplementation. Densitometric data of band analysis are presented in Figure 4.
